# 4-Amino-1*H*-1,2,4-triazol-1-ium nitrate

**DOI:** 10.1107/S1600536810049949

**Published:** 2010-12-04

**Authors:** Irena Matulková, Ivana Císařová, Ivan Němec

**Affiliations:** a Department of Inorganic Chemistry, Faculty of Science, Charles University in Prague, Hlavova 2030, 128 40 Prague 2, Czech Republic; b Department of Spectroscopy, J. Heyrovský Institute of Physical Chemistry, ASCR, v.v.i., Dolejškova 3, 182 23 Prague 8, Czech Republic

## Abstract

The non-centrosymmetric crystal structure of the novel semi-organic title compound, C_2_H_5_N_4_^+^·NO_3_^−^, is based on alternating layers of 4-amino-1*H*-1,2,4-triazolinium cations (formed by parallel chains of cations mediated by weak C—H⋯N hydrogen bonds) and nitrate anions inter­connected *via* linear and bifurcated N—H⋯O hydrogen bonds and weak C—H⋯O hydrogen bonds. N—H⋯N hydrogen bonds link the anions and cations.

## Related literature

For the uses of triazole complexes in medicine, see: Li *et al.* (2004 ▶); Komeda *et al.* (2003 ▶); Mernari *et al.* (1998 ▶); Bentiss *et al.* (1999 ▶). For the triazole moiety as a part of the ligand system in metal complexes, see: Sinditskii *et al.* (1987 ▶); Haasnoot (2000 ▶); Klingele & Brooker (2003 ▶); Beckmann & Brooker (2003 ▶); Muller *et al.* (2003 ▶). For the non-linear optical properties of 4-amino-1,2,4-triazole or 3-amino-1,2,4-triazoles, see: Matulková *et al.* (2007 ▶, 2008 ▶). For the preparation of 4-amino-1,2,4-triazole, see: Herbert & Garrison (1953 ▶); Matulková *et al.* (2008 ▶); Sanz *et al.* (2002 ▶). 
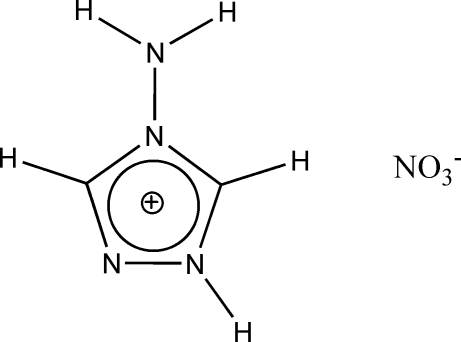


## Experimental

### 

#### Crystal data

C_2_H_5_N_4_^+^·NO_3_^−^
*M* *_r_* = 147.11
Monoclinic, 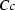

*a* = 9.6200 (9) Å
*b* = 5.2790 (3) Å
*c* = 11.895 (1) Å
β = 96.667 (3)°
*V* = 599.99 (8) Å^3^
*Z* = 4
Mo *K*α radiation
μ = 0.15 mm^−1^
*T* = 293 K
0.5 × 0.4 × 0.35 mm

#### Data collection

Nonius KappaCCD area-detector diffractometer
1867 measured reflections
685 independent reflections
650 reflections with *I* > 2σ(*I*)
*R*_int_ = 0.026

#### Refinement

*R*[*F* ^2^ > 2σ(*F* ^2^)] = 0.027
*wR*(*F* ^2^) = 0.073
*S* = 1.11
685 reflections
92 parameters
2 restraints
H-atom parameters constrained
Δρ_max_ = 0.12 e Å^−3^
Δρ_min_ = −0.14 e Å^−3^

### 

Data collection: *COLLECT* (Hooft, 1998 ▶) and *DENZO* (Otwin­owski & Minor, 1997 ▶); cell refinement: *COLLECT* and *DENZO*; data reduction: *COLLECT* and *DENZO*; program(s) used to solve structure: *SIR92* (Altomare *et al.*, 1994 ▶); program(s) used to refine structure: *SHELXL97* (Sheldrick, 2008 ▶); molecular graphics: *PLATON* (Spek, 2009 ▶); software used to prepare material for publication: *SHELXL97*.

## Supplementary Material

Crystal structure: contains datablocks I, global. DOI: 10.1107/S1600536810049949/zq2079sup1.cif

Structure factors: contains datablocks I. DOI: 10.1107/S1600536810049949/zq2079Isup2.hkl

Additional supplementary materials:  crystallographic information; 3D view; checkCIF report

## Figures and Tables

**Table 1:** Hydrogen-bond geometry (Å, °)

*D*—H⋯*A*	*D*—H	H⋯*A*	*D*⋯*A*	*D*—H⋯*A*
N2—H2⋯O1^i^	0.89	1.83	2.710 (2)	173
N2—H2⋯N7^i^	0.89	2.53	3.315 (2)	148
N2—H2⋯O2^i^	0.89	2.54	3.086 (2)	120
N6—H6*A*⋯O3	0.96	2.22	3.077 (3)	148
N6—H6*B*⋯O3^ii^	0.95	2.30	3.008 (3)	131
N6—H6*B*⋯O1^ii^	0.95	2.45	3.112 (3)	126
N6—H6*B*⋯O2^iii^	0.95	2.59	3.167 (3)	119
C3—H3⋯N1^iii^	0.93	2.45	3.299 (3)	151
C5—H5⋯O2	0.93	2.55	3.398 (3)	153

Symmetry codes: (i) 
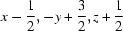
; (ii) 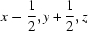; (iii) 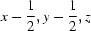.

**Fig. 1.**
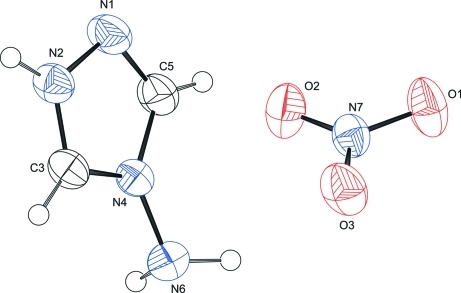
The atom-labelling of the title compound. Displacement ellipsoids are drawn at the 50% probability level.

**Fig. 2.**
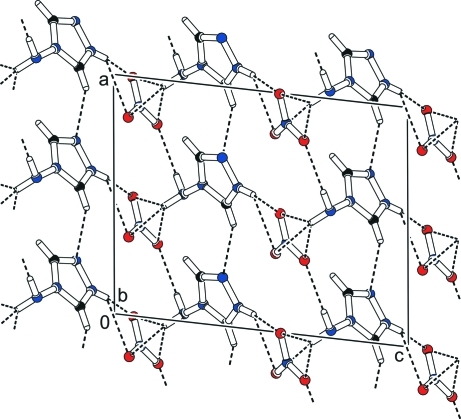
A packing scheme of the title compound (projection to ac plane). Hydrogen bonds are indicated by dashed lines (C—H···O bonds are omitted for clarity).

## supplementary crystallographic information

### Comment

The 1,2,4-triazole moiety as a part of ligand system in metal complexes has gained considerable attention in recent years (Sinditskii *et al.*, 1987; Haasnoot, 2000; Klingele & Brooker, 2003; Beckmann & Brooker, 2003; Muller *et al.*, 2003). The application of triazole ligand lies in the medical research – complex with Pt^II^ (Komeda *et al.*, 2003) exhibits antitumor activity. Triazole derivatives are also used in the synthesis of antibiotics, fungicides, herbicides, plant growth hormone regulators (Li *et al.*, 2004), and potentially prospective corrosion inhibitors (Mernari *et al.*, 1998; Bentiss *et al.*, 1999).

Materials based on triazole compounds with dicarboxylic acids (4-amino-1,2,4-triazol-1-ium hydrogen oxalate, adducts of 4-amino-1,2,4-triazole with succinic acid and adipic acid and 3-amino-1,2,4-triazolinium hydrogen *L*-tartrate) were also prepared and characterized as promising compounds in the field of non-linear optics (Matulková *et al.*, 2008; Matulková *et al.*, 2007).

The non-centrosymmetric crystal structure of the title compound is based on alternating layers of 4-amino-1,2,4-triazolinium cations (formed by parallel chains of cations mediated by weak C—H···N hydrogen bonds) and nitrate anions inter-connected *via* linear and bifurcated N—H···O hydrogen bonds and weak C—H···O hydrogen bond. The donor···acceptor distances in these hydrogen bonds attain values from 2.710 (2) to 3.167 (3) Å (N—H···O bonds) and 3.398 (3) Å (C—H···O bond). The Fig. 1 contains atom-labelling of title compound and packing scheme of the crystal structure is presented in Fig. 2.

The bond length comparison of 4-amino-1,2,4-triazolinium cations in the title compound and 4-amino-1,2,4-triazol-1-ium hydrogen oxalate (Matulková *et al.*, 2008) exhibits reasonable shortening of N1—N2 distance in the cation of the title compound compared to N2—N3 distance in the organic salt (1.359 (2) Å for 4-amino-1,2,4-triazol-1-ium nitrate; 1.366 (2) Å for 4-amino-1,2,4-triazol-1-ium hydrogen oxalate).

The nitrate anions are planar with O—N—O angles slightly different from theoretical values of 120° in the crystal structure of the title compound. These differences can be explained by unequal participation of oxygen atoms in N—H···O hydrogen bonds. The smaller angle values 119.7 (2) and 118.5 (2)° (*i.e.* angles O1—N7—O2 and O3—N7—O1) are connected with the existence of bifurcated hydrogen bonds influencing these parts of the anion. The remaining bond angle O2—N7—O3 shows a value of 121.8 (2)°. Similar differences can be also observed in the case of N—O bond distances - *i.e.* two short distances 1.232 (3) and 1.240 (2) Å (N7—O2 and N7—O3, respectively) and a longer one 1.263 (2) Å (N7—O1).

### Experimental

4-Amino-1,2,4-triazole was prepared and purified by a slightly modified procedure described previously in the literature (Sanz *et al.*, 2002; Herbert *et al.*, 1953; Matulková *et al.*, 2008). The crystals of the title compound, were obtained from a solution of 0.1 g of 4-amino-1,2,4-triazole, 0.8 ml of 2 mol/dm^3^ nitric acid (68% p.a., Lachema) and 5 ml of water. The solution was left to crystallize at room temperature for several weeks. The colourless crystals obtained were filtered off, washed with methanol and dried in vacuum desiccator over KOH.

### Refinement

H atoms attached to C atoms were calculated in geometrically idealized positions, with C(*sp*^2^)—H = 0.93 Å. The positions of H atoms attached to O and N atoms were localized on difference Fourier maps. All hydrogen atoms were constrained to ride on their parent atoms during refinement, with *U*_iso_(H) = 1.2 *U*_eq_ (pivot atom).

### Crystal data

**Table:**

C_2_H_5_N_4_^+^·NO_3_^−^	*F*(000) = 304
*M**_r_* = 147.11	*D*_x_ = 1.629 Mg m^−^^3^
Monoclinic, *C**c*	Mo *K*α radiation, λ = 0.71073 Å
Hall symbol: C -2yc	Cell parameters from 572 reflections
*a* = 9.6200 (9) Å	θ = 1.0–27.5°
*b* = 5.2790 (3) Å	µ = 0.15 mm^−^^1^
*c* = 11.895 (1) Å	*T* = 293 K
β = 96.667 (3)°	Prism, colourless
*V* = 599.99 (8) Å^3^	0.5 × 0.4 × 0.35 mm
*Z* = 4	

### Data collection

**Table:**

Nonius KappaCCD area-detector diffractometer	650 reflections with *I* > 2σ(*I*)
Radiation source: fine-focus sealed tube	*R*_int_ = 0.026
graphite	θ_max_ = 27.5°, θ_min_ = 3.5°
Detector resolution: 9.091 pixels mm^-1^	*h* = −11→12
φ and ω scans to fill the Ewald sphere	*k* = −5→6
1867 measured reflections	*l* = −15→15
685 independent reflections	

### Refinement

**Table:**

Refinement on *F*^2^	Secondary atom site location: difference Fourier map
Least-squares matrix: full	Hydrogen site location: difference Fourier map
*R*[*F*^2^ > 2σ(*F*^2^)] = 0.027	H-atom parameters constrained
*wR*(*F*^2^) = 0.073	*w* = 1/[σ^2^(*F*_o_^2^) + (0.0382*P*)^2^ + 0.111*P*] where *P* = (*F*_o_^2^ + 2*F*_c_^2^)/3
*S* = 1.11	(Δ/σ)_max_ < 0.001
685 reflections	Δρ_max_ = 0.12 e Å^−^^3^
92 parameters	Δρ_min_ = −0.14 e Å^−^^3^
2 restraints	Extinction correction: *SHELXL97* (Sheldrick, 2008), Fc^*^=kFc[1+0.001xFc^2^λ^3^/sin(2θ)]^-1/4^
Primary atom site location: structure-invariant direct methods	Extinction coefficient: 0.104 (8)

### Special details

**Table:**

Geometry. All e.s.d.'s (except the e.s.d. in the dihedral angle between two l.s. planes) are estimated using the full covariance matrix. The cell e.s.d.'s are taken into account individually in the estimation of e.s.d.'s in distances, angles and torsion angles; correlations between e.s.d.'s in cell parameters are only used when they are defined by crystal symmetry. An approximate (isotropic) treatment of cell e.s.d.'s is used for estimating e.s.d.'s involving l.s. planes.
Refinement. Refinement of *F*^2^ against ALL reflections. The weighted *R*-factor *wR* and goodness of fit *S* are based on *F*^2^, conventional *R*-factors *R* are based on *F*, with *F* set to zero for negative *F*^2^. The threshold expression of *F*^2^ > σ(*F*^2^) is used only for calculating *R*-factors(gt) *etc*. and is not relevant to the choice of reflections for refinement. *R*-factors based on *F*^2^ are statistically about twice as large as those based on *F*, and *R*- factors based on ALL data will be even larger.

### Fractional atomic coordinates and isotropic or equivalent isotropic displacement parameters (Å^2^)

**Table:**

	*x*	*y*	*z*	*U*_iso_*/*U*_eq_	
N1	0.20255 (19)	0.8902 (4)	0.36897 (16)	0.0454 (5)	
N2	0.08599 (18)	0.8575 (3)	0.42181 (14)	0.0378 (4)	
H2	0.0630	0.9678	0.4730	0.045*	
C3	0.0132 (2)	0.6606 (4)	0.38252 (16)	0.0370 (4)	
H3	−0.0698	0.6022	0.4062	0.044*	
N4	0.08113 (16)	0.5606 (3)	0.30229 (14)	0.0337 (4)	
C5	0.1957 (2)	0.7065 (5)	0.29519 (19)	0.0436 (5)	
H5	0.2608	0.6793	0.2444	0.052*	
N6	0.0325 (2)	0.3489 (3)	0.23683 (17)	0.0424 (4)	
H6A	0.1058	0.2463	0.2131	0.051*	
H6B	−0.0266	0.4027	0.1716	0.051*	
N7	0.37711 (17)	0.3656 (4)	0.08773 (13)	0.0383 (4)	
O1	0.49649 (17)	0.2961 (4)	0.06543 (15)	0.0563 (5)	
O2	0.3276 (2)	0.5676 (3)	0.04961 (17)	0.0555 (5)	
O3	0.31256 (18)	0.2262 (4)	0.14762 (15)	0.0566 (5)	

### Atomic displacement parameters (Å^2^)

**Table:**

	*U*^11^	*U*^22^	*U*^33^	*U*^12^	*U*^13^	*U*^23^
N1	0.0350 (9)	0.0525 (10)	0.0509 (10)	−0.0089 (8)	0.0142 (8)	−0.0060 (8)
N2	0.0347 (8)	0.0442 (9)	0.0359 (8)	−0.0036 (7)	0.0096 (6)	−0.0009 (7)
C3	0.0324 (10)	0.0446 (11)	0.0350 (9)	−0.0038 (8)	0.0088 (7)	0.0027 (8)
N4	0.0301 (8)	0.0366 (9)	0.0346 (7)	−0.0019 (6)	0.0052 (6)	0.0008 (6)
C5	0.0345 (10)	0.0488 (12)	0.0497 (12)	−0.0039 (9)	0.0147 (9)	−0.0048 (9)
N6	0.0425 (10)	0.0374 (9)	0.0472 (9)	−0.0025 (7)	0.0049 (7)	−0.0050 (7)
N7	0.0354 (10)	0.0448 (10)	0.0354 (8)	0.0046 (7)	0.0067 (7)	−0.0015 (7)
O1	0.0444 (10)	0.0686 (11)	0.0597 (10)	0.0175 (9)	0.0221 (8)	0.0214 (8)
O2	0.0570 (10)	0.0488 (10)	0.0627 (10)	0.0157 (8)	0.0152 (8)	0.0068 (8)
O3	0.0458 (9)	0.0649 (11)	0.0623 (11)	0.0013 (9)	0.0202 (8)	0.0160 (9)

### Geometric parameters (Å, °)

**Table:**

N1—C5	1.304 (3)	N4—N6	1.411 (2)
N1—N2	1.359 (2)	C5—H5	0.9300
N2—C3	1.309 (3)	N6—H6A	0.9572
N2—H2	0.8888	N6—H6B	0.9506
C3—N4	1.327 (3)	N7—O2	1.232 (3)
C3—H3	0.9300	N7—O3	1.240 (2)
N4—C5	1.355 (3)	N7—O1	1.263 (2)
			
C5—N1—N2	103.71 (17)	N1—C5—N4	111.00 (18)
C3—N2—N1	111.81 (18)	N1—C5—H5	124.5
C3—N2—H2	126.9	N4—C5—H5	124.5
N1—N2—H2	121.2	N4—N6—H6A	113.8
N2—C3—N4	106.57 (17)	N4—N6—H6B	110.0
N2—C3—H3	126.7	H6A—N6—H6B	108.6
N4—C3—H3	126.7	O2—N7—O3	121.80 (19)
C3—N4—C5	106.89 (17)	O2—N7—O1	119.72 (19)
C3—N4—N6	123.56 (17)	O3—N7—O1	118.48 (19)
C5—N4—N6	129.48 (18)		

### Hydrogen-bond geometry (Å, °)

**Table:**

*D*—H···*A*	*D*—H	H···*A*	*D*···*A*	*D*—H···*A*
N2—H2···O1^i^	0.89	1.83	2.710 (2)	173.
N2—H2···N7^i^	0.89	2.53	3.315 (2)	148.
N2—H2···O2^i^	0.89	2.54	3.086 (2)	120.
N6—H6A···O3	0.96	2.22	3.077 (3)	148.
N6—H6B···O3^ii^	0.95	2.30	3.008 (3)	131.
N6—H6B···O1^ii^	0.95	2.45	3.112 (3)	126.
N6—H6B···O2^iii^	0.95	2.59	3.167 (3)	119.
C3—H3···N1^iii^	0.93	2.45	3.299 (3)	151.
C5—H5···O2	0.93	2.55	3.398 (3)	153.

Symmetry codes: (i) *x*−1/2, −*y*+3/2, *z*+1/2; (ii) *x*−1/2, *y*+1/2, *z*; (iii) *x*−1/2, *y*−1/2, *z*.
